# Symptomatic hemiparkinsonism due to extensive middle and posterior fossa arachnoid cyst: case report

**DOI:** 10.1186/s12883-020-01670-y

**Published:** 2020-03-12

**Authors:** Bernadette Wimmer, Stephanie Mangesius, Klaus Seppi, Sarah Iglseder, Franziska Di Pauli, Martin Ortler, Elke Gizewski, Werner Poewe, Gregor Karl Wenning

**Affiliations:** 1 Department of Neurology, Medical University Innsbruck, Anichstrasse 35, Innsbruck, Austria; 2 Department of Neurology, Medical University Regensburg, Universitätsstraße 84, 93053 Regensburg, Germany; 3 Department of Neuroradiology, Medical University Innsbruck, Anichstrasse 35, Innsbruck, Austria; 4 Neuroimaging Core Facility, Medical University Innsbruck, Anichstrasse 35, Innsbruck, Austria; 5 Department of Neurosurgery, Medical University Innsbruck, Anichstrasse 35, Innsbruck, Austria

**Keywords:** Fenestration, Brainstem, Basal ganglia

## Abstract

**Introduction:**

Intracranial neoplasms are an uncommon cause of symptomatic parkinsonism. We here report a patient with an extensive middle and posterior fossa arachnoid cyst presenting with parkinsonism that was treated by neurosurgical intervention.

**Methods:**

Retrospective chart review and clinical examination of the patient.

**Case report:**

This 55-year-old male patient with hemiparkinsonism and recurrent bouts of headaches was first diagnosed in 1988. CT scans revealed multiple cystic lesions compressing brainstem and basal ganglia, which were partially resected. Subsequently, the patient was free of complaints for 20 years. In 2009 the patient presented once more with severe unilateral tremor and thalamic pain affecting the right arm. Despite symptomatic treatment with L-Dopa and pramipexole symptoms worsened over time. In 2014 there was further progression with increasing hemiparkinsonism, hemidystonia, unilateral thalamic pain and pyramidal signs. Repeat CT scanning revealed a progression of the cysts as well as secondary hydrocephalus. Following repeat decompression of the brainstem and fenestration of all cystic membranes parkinsonism improved with a MDS- UPDRS III score reduction from 39 to 21. Histology revealed arachnoid cystic material.

**Conclusion:**

We report on a rare case of recurrent symptomatic hemiparkinsonism resulting from arachnoid cysts.

## Background

Arachnoid cysts are constituted of fluid collections within the central nervous system, which commonly remain asymptomatic. Occasional symptomatic forms are described to result in hemiparkinsonism [1–3]. A conservative treatment approach is generally recommended, however, in symptomatic cases a timely neurosurgical intervention can still be indicated [4, 5]. The aim of this case report is to illustrate an unusual cause of symptomatic hemiparkinsonism. We here report a patient with an extensive arachnoid cyst presenting with hemiparkinsonism and thalamic pain improving by repeated neurosurgical decompression.

## Case presentation

**Fig. 1:**
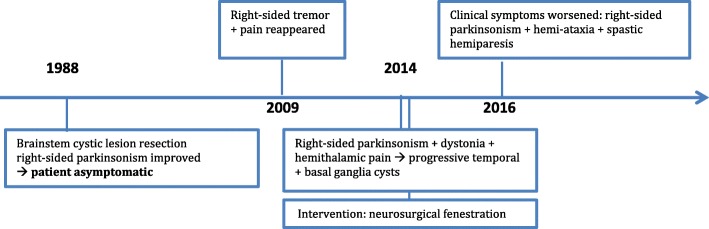
Timeline of important information of the patient’s history

A 29-year-old male patient complained about recurrent bouts of headaches. In the following month he developed a progressive right-sided slowness, which limited his ability to work. On admission to hospital in 1988 he was diagnosed with strictly right sided parkinsonism, however, the symptoms were only partially responsive to levodopa. CT scans revealed multiple cystic lesions within the brainstem as well as a posterior cerebral artery aneurysm. Due to the severity of the motor symptoms as well as the indication of surgical treatment for the aneurysm, the patient underwent neurosurgical intervention with partial resection of the cystic lesions and aneurysm clipping at an outside institution. Subsequently, the patient was asymptomatic for 20 years and able to pursue his former work without any physical limitations. In 2009 the patient presented again with chronically progressive unilateral tremor and thalamic pain affecting the right arm. DAT-SPECT revealed severe strictly unilateral dopaminergic denervation on the left side. Despite symptomatic treatment with escalating dosages of levodopa and pramipexole, symptoms worsened over time (Fig. 1).

Clinical examination in August 2014 revealed further progression with increasing right-sided hemiparkinsonism, hemidystonia, hemithalamic pain as well as pyramidal signs. The patient was severely impaired in his activities of daily living and was suffering from mild depression. Repeated CT scanning showed progressive temporo-mesial and basal ganglia cysts as well as the development of a secondary hydrocephalus. As posterior cerebral artery aneurysm clipping was performed in the 90ies, magnetic resonance imaging to determine the exact configuration of the intracerebral cysts was contraindicated. Digital subtraction angiography revealed an aneurysm recurrence. In an open microsurgical procedure, fenestration of cystic membranes was performed, whereas full exploration of the aneurysm was abolished because of the limited access and the potential morbidity associated with a dissection within the severely scarred tissue. Parkinsonian signs, as measured by the MDS Unified Parkinson’s Disease Rating Scale (MDS-UPDRS) part III, improved with a score reduction from 39 (pre-operatively) to 21 (postoperatively), showing a very good response of motor symptoms to the treatment. The pre and post-operation video assessment is included in the supplementary file. Concerning the non-motor symptoms, thalamic pain as well as depression improved markedly. The follow-up CT scan revealed no significant volume change of the cystic lesions Neuropathological examination revealed features of arachnoid cysts.


**Additional file 1** Pre-operative assessment (18/11/2014) In this video the patient is shown before surgery presenting with a marked gait dysfunction and instability. Post-operative assessment (02/12/2014) The patient is shown at day 12 after surgery. Parkinsonian symptoms are markedly improved. The patient now exhibits a slight hemiparesis due to cystic lesions, which improved over time.


At last follow-up in April 2016 the patient marginally deteriorated again on a daily dose of 1200 mg of levodopa. He reported motor fluctuations with shuffling of gait, increasing rigidity, nervousness and dystonia during Off-periods. Neurological examination revealed secondary right-sided hemiparkinsonism with superimposed hemi-ataxia, and spastic hemiparesis, although repeated CT scans demonstrated no significant morphological change.

## Discussion and conclusion

Secondary parkinsonism caused by arachnoid cysts is rarely described [1–3]. Arachnoid cysts usually represent incidental findings of fluid collections in the central nervous system without clinical relevance, for which a conservative treatment approach is generally recommended [4].

In contrast, invasive treatment strategies can be considered for symptomatic arachnoid cysts, as they may cause multiple impairments such as headache, paresis, parkinsonism, seizures, hydrocephalus, cognitive decline and visual loss [5], with headache representing the most frequent symptom in up to 50%.

The optimal surgical treatment option for such lesions is debated [5–7]. Recent advances in neurosurgery offer several treatment options, such as shunt placement, craniotomy, endoscopic fenestration, and stereotactic aspiration. A recent case series showed fenestration to be highly effective [5]. Since inspection and reclipping of the recurrent aneurysm was deemed necessary, our patient was treated with microsurgical fenestration. This resulted in a marked improvement of motor features estimated by means of MDS-UPDRS III after surgery. Despite the marked clinical improvement, the morphological configuration of the cysts estimated by CT scans was just marginally reduced. Reason for this finding might be the limited resolution of the CT scans, which might lead to an underestimation of the cystic extent. Magnetic resonance imaging might give more detailed morphological information; however, this imaging modality could not be applied to our patient.

Various cortical and subcortical lesions are known to cause parkinsonism [8] and there is often a huge discrepancy between lesion load and clinical findings [9] so that no clear correlation between lesion localization and clinical findings can be established. In our patient the initial lesions were located within the brainstem, whereas the later clinical symptoms where due to cystic lesions within the basal ganglia. Medical response as well as effective brain stimulation is thought to depend on the functional connectivity of the lesion to the basal ganglia and the midbrain. As such, a change in functional connectivity might also lead to clinical improvement [8].

Cyst recurrences after surgery were thought to be rare [5] as was shown in a case series were only 3 out of 20 patients repeatedly developed cystic relapses. However, this might be explained by the short observational period of four years in this study. In our case the follow-up period was over 20 years, with the cystic lesions reappearing after more than 10 years, which were subsequently efficiently treated by a second surgical intervention, showing a marked improvement of the MDS-UPDRS with more than 30 % reduction of the total motor score, which is equally effective as levodopa in idiopathic Parkinson’s disease patients. Even two years after the surgical treatment, the parkinsonian symptoms showed an improvement, despite there being a slight worsening over the follow-up period of two years. Potential reasons for the reoccurrence of symptoms are various and might be attributed to age, surgical pathway or reorganisation of cysts, despite there being no morphological evidence for that. We therefore conclude that cerebral arachnoid cysts need a close and long-term follow up. Furthermore, surgical intervention can be seen as a treatment option also in case of relapse.

**Fig. 2:**
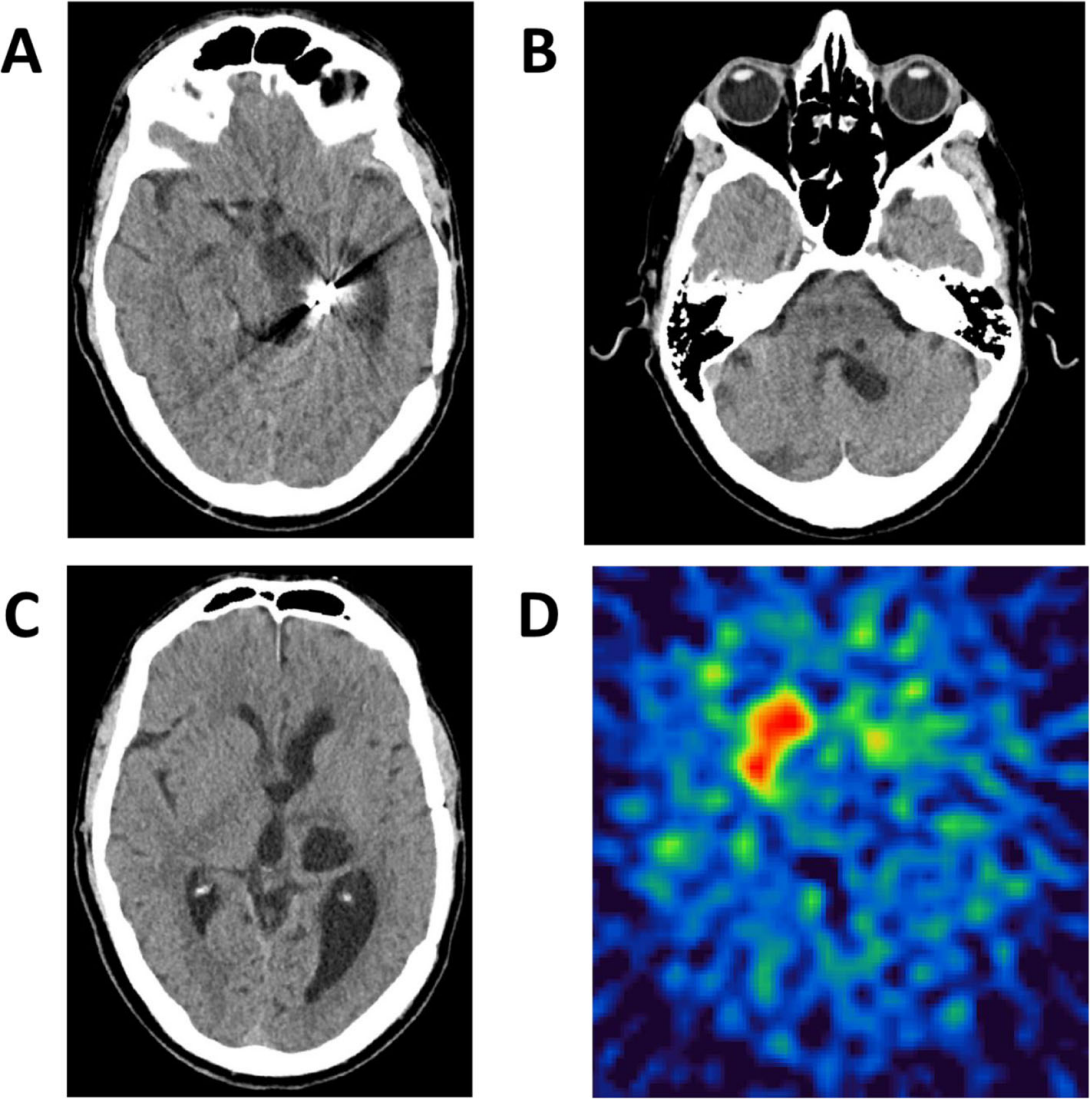
Arachnoid cysts in a patient suffering from secondary right-sided hemiparkinsonism with superimposed hemiataxia and spastic hemiparesis. CCT-scans showing (**a**) the aneurysma clip, (**b**) pons, (**c**) cystic lesions and ventricular enlargement, as well as (**d**) DAT-SPECT imaging showing severe striatal dopaminergic denervation on the left side. Also note the artefacts for the previously clipped aneurysm

Patients suffering from symptomatic parkinsonism may exhibit all types of parkinsonian signs including akinesia, rigidity and tremor [1]. However, levodopa response is often reduced in patients with vascular as well as space-occupying lesions [10]. In our case, parkinsonian signs were clearly related to severe and strictly unilateral dopaminergic denervation of the striatum as shown by dopamine transporter SPECT imaging (see Fig. 2). The inadequate response to levodopa reflected loss of dopamine receptors due to extension of cystic lesions into the basal ganglia, as lesions within the basal ganglia itself predict a poor levodopa responsiveness [11]. Additional clinical features such as contralateral pyramidal sign, hemihypesthesia and thalamic pain were associated with cystic disruption of respective pathways.

We here report a patient with symptomatic levodopa-refractory hemiparkinsonism resulting from extensive arachnoid cystic lesions disrupting contralateral basal ganglia. Neurosurgical decompression resulted in extended periods of remission or improvement in this patient, however, future studies are needed to determine the effectiveness of this treatment.

## Data Availability

Data sharing is not applicable to this article as no datasets were generated or analysed during the current study.

## Abbreviations

CT: Computed Tomography
DAT SPECT: Dopamine Transporter Single-photon emission computed tomography
MDS UPDRS: Movement Disorder Society Unified Parkinson’s Disease Rating Scale
